# Evaluating quackery formulations: LC-MS/MS based method for detecting glucocorticoid content

**DOI:** 10.1016/j.toxrep.2024.101763

**Published:** 2024-10-09

**Authors:** Hafiza Monaza Batool, Muhammad Irfan Jalees, Madeeha Batool

**Affiliations:** a School of Chemistry, University of the Punjab, Lahore 54590, Pakistan; b Institute of Environmental Engineering and Research, University of Engineering and Technology, Lahore 54890, Pakistan

**Keywords:** Liquid chromatography-tandem mass spectrometry (LC-MS/MS), Glucocorticoids, Quackery formulations, Method development, Validation

## Abstract

Glucocorticoids are widely used as highly effective drugs for treating inflammatory diseases. In this study, a method was developed and validated using liquid chromatography-tandem mass spectrometry (LC-MS/MS) to simultaneously determine four glucocorticoids, including betamethasone, dexamethasone, hydrocortisone, and prednisolone in unauthorized or unregulated medicinal powders often associated with quackery formulations. Commercially available standards were used for method development and glucocorticoid detection. Glucocorticoids were extracted from the samples with methanol, which were then chromatographically separated using two mobile phases (0.1 % formic acid in water and 0.1 % formic acid in acetonitrile) in an isocratic flow on an Agilent Poroshel 120 C18 column (2.1 mm x 75 mm x 2.7 m). The validated analytical measuring range (AMR) of betamethasone and dexamethasone was 7.8–500 ng/mL, whereas, for hydrocortisone and prednisolone, AMR was 7.8–1000 ng/mL. The method showed an excellent coefficient of determination (r2) >0.990 for betamethasone, hydrocortisone, and prednisolone, while for dexamethasone 0.986. Accuracy and precision (intra/inter days) of these glucocorticoids showed a bias of 6–15 % (<20 %) and a coefficient of variation (CV) of <15 %. For each dilution factor, the integrity of samples was maintained after dilution. The developed method is sensitive and valuable for detecting, quantifying, and confirming the selected glucocorticoids in various quackery formulation powders commonly used in Pakistani setups.

## Highlights

• An LC-MS/MS method for simultaneous determination of Glucocorticoids.
• Quantification of Betamethasone, Dexamethasone, Hydrocortisone, and Prednisolone.
• A highly sensitive method for simultaneous detection of glucocorticoid contents.
• A practical tool to screen Glucocorticoid contents in Quacks formulating powders in Pakistan.

## Graphical Abstract

**Figure:**
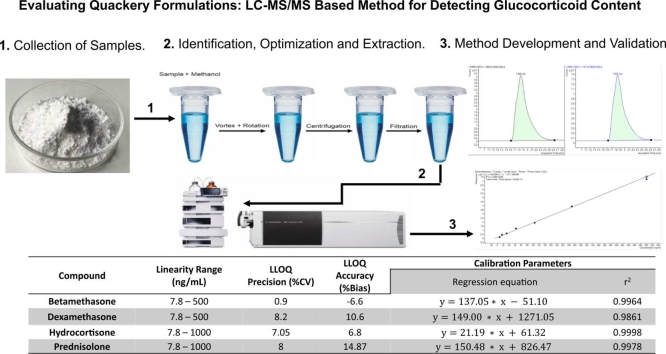

## 1 Introduction

Glucocorticoids, a category of steroid hormones, possess a strong affinity for glucocorticoid receptors distributed throughout the human body [1]. These receptors are integral to the endocrine system and have pivotal roles in various physiological processes. Binding between glucocorticoid and its receptors activates the effects of these hormones either slowly via nuclear receptors or rapidly via non-genomically mediated membrane-associated receptors [2]. Due to their potential antiangiogenic, anti-edematous, and antiapoptotic properties, synthetic glucocorticoids have been widely reported to treat inflammation and autoimmune diseases, such as arthritis, allergies, dermatological disorders, and various cancers [3]. Some commonly used glucocorticoids include betamethasone, dexamethasone, hydrocortisone, and prednisolone [4]. These glucocorticoid-based drugs are structurally related to cortisol, which is a naturally occurring glucocorticoid in humans [5]; however, they differ in their pharmacokinetic and pharmacodynamic properties [6]. Unfortunately, their non-judicious use in high doses harms human health [7], [8]. Prolonged therapy with glucocorticoid preparations can lead to adverse effects, such as cutaneous reactivity, skin atrophy, and systemic side effects, including osteoporosis, diabetes mellitus, Cushing's syndrome, allergic contact dermatitis, hypertension, and increased susceptibility to infections [4], [9].

Various types of glucocorticoids are found in different materials, such as cosmetics, dietary supplements, quackery formulations, pharmaceutical drugs, and more [10]. Quackery formulations refer to remedies or products that are promoted as effective treatments without scientific backing, often making exaggerated or false health claims. In countries like Pakistan, where access to quality healthcare is limited, people may resort to such formulations out of desperation, affordability, or lack of awareness about their potential dangers. Quacks, who are uncertified and self-proclaimed healthcare providers, exploit this situation by preparing and distributing these unregulated formulations without proper knowledge of the ingredients or their effects [11].

People prefer the drugs prescribed by quacks for various diseases because of several factors, including cultural beliefs, cheaper rates, and over-the-counter availability [12], [13]. According to popular public opinion, these powders are regarded as somewhat of a magical medication that can alleviate a patient's symptoms in a very short duration [14]. However, their long-term or frequent use can cause many physical and physiological pathologies in humans [15]. Generally, for analysis, two types of assays are used for GC. One of them is antibody analytes that include radioimmunoassay (RIA), enzyme immunoassay (EIA), chemiluminescence, and bead-based luminescent amplification assays [16]. The other one is direct quantification using techniques such as HPLC and LC-MS/MS.

Previously, different techniques such as HPLC and LC-MS/MS have been reported for the analysis of glucocorticoids in biological fluids [17], pharmaceutical and cosmetic products [18], and urine [19], [20]. Subsequently, LC-MS /MS proves itself as a new tool to evaluate these steroids in human matrices such as adipose tissues [21], hair [22], cortisone in human scalp hair [23], and milk and plasma [24]. The LC-MS/MS technique, with different conditions, has been successfully applied to liquid-based samples such as cosmetics and other human fluids; they are not optimized for direct analysis of glucocorticoids in powdery formulations.

**Fig. 1:**
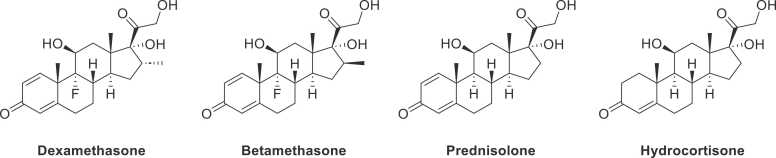
Chemical structures of glucocorticoids used in this study.

Keeping in view the extensive network of quacks prevailing in our country and the number of people who get treated by them on a regular basis [25], there was a dire need to develop a method for assessing the presence of commonly used glucocorticoids in these powders, focusing on accuracy and precision as per US Food and Drug Administration (FDA) guidelines (2018 guidance for industry) [26], [27]. By focusing on the unique properties of powders rather than applying a standard liquid-based approach, with this study, we provided a validated method that can be performed with a simplified approach and has good sensitivity. Four selected glucocorticoids have been identified and quantified, that is betamethasone, dexamethasone, hydrocortisone, and prednisolone in quackery formulation powders. (Fig. 1) With the development of this method, we intended to highlight the dangers which are associated with the usage of quackery formulations and the harmful ingredients they are comprised of.

## 2 Material and methods

### 2.1 Standards and chemicals

Glucocorticoids standards, including Betamethasone (CAS Number: PHR1398 vial containing 1 g), Dexamethasone (CAS Number: D4902 vial containing 100 mg), Hydrocortisone (CAS Number: 50–23–7 vial containing 1 g), and Prednisolone (CAS Number: PHR1043 vial containing 500 mg), in powdered form, were purchased from Sigma-Aldrich. The purity of glucocorticoid standards was found to be in the range of 99–95 %. None of the standards had a purity of less than 95 %. For sample extraction and mobile phase preparation, analytical grade chemicals were used. Formic acid (99 %, w/w) was obtained from Sigma-Aldrich, and methanol and acetonitrile (LC-MS/MS grade) were obtained from Merck. Ultrapure water (Millipore Merck, Germany) was used for all the experimental procedures.

### 2.2 Preparation of stock solutions of standards

Standard stock solution (A) of 1000 µg/mL concentration was prepared by dissolving 10 mg of commercially available dexamethasone in methanol [1], [4] using a 10 mL volumetric flask. Its volume was brought up to 10 mL with methanol. From standard stock solution (A), 100 µL was transferred into a 10 mL volumetric flask, and their volume was brought up to 10 mL with methanol to prepare a standard stock solution (B) of 10 µg/mL concentration. From (B), 1 mL was transferred into a 10 mL volumetric flask to make a working standard stock solution (C), and its volume was brought up to 10 mL with methanol, giving a concentration of 1 µg/mL or 1000 ng/mL. Similarly, Standard Stock Solutions (A), (B), and (C) of Betamethasone, hydrocortisone, and prednisolone were prepared.

### 2.3 Preparation of working standards (Calibrators)

Eight calibrators were prepared from the 1000 ng/mL Standard Stock Solutions (C) of Betamethasone, Dexamethasone, Hydrocortisone, and Prednisolone through serial dilution [28]. Each of the eight GC vials was labeled with the corresponding concentrations: 1000 ng/mL, 500 ng/mL, 250 ng/mL, 125 ng/mL, 62.5 ng/mL, 31.25 ng/mL, 15.625 ng/mL, and 7.8 ng/mL. Initially, 1000 µL of the Standard Stock Solution was transferred to the vial labeled 1000 ng/mL, while the remaining vials were filled with 500 µL of pure methanol. Serial dilution was then performed by transferring 500 µL from the higher-concentration vial to the next, beginning with the 1000 ng/mL vial and continuing down to the 7.8 ng/mL vial, with vortexing for 30 seconds between each step to ensure thorough mixing. This procedure was repeated for all four analytes.

### 2.4 Preparation of sample solutions

**Fig. 2:**
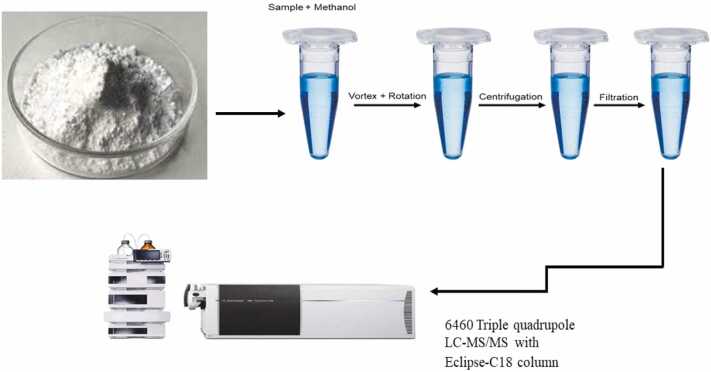
Schematic representation of the sample preparation process for LC-MS/MS analysis**.**

One gram of quackery formulation powder was dissolved in 4 mL methanol in a sample vial. The sample vial was vortexed for 30 seconds to ensure mixing. The mixed sample was then swirled for 30 minutes using a rotator at 30 RPM, followed by centrifugation at 3000 RPM for 10 minutes. The supernatant was aspirated in a syringe and filtered into an injection vial through a 0.2 micron syringe filter (Despite the absence of visible precipitates in the homogenized solution, centrifugation and filtration were conducted as per safety manual requirements for ensuring sample purity essential for accurate LC-MS/MS analysis). It was then injected into LC-MS/MS 6460 Agilent Technologies, USA. The samples were processed and analyzed in triplicate to ensure reproducibility. (Fig. 2).

### 2.5 Instrumentation

#### 2.5.1 Chromatographic conditions

The analytical method used was high-performance liquid chromatography (HPLC), Agilent 1260 infinity series combined with triple quadrupole mass spectrometry, and Agilent 6460 Triple Quad QQQ. A 10 µL sample was taken for injection. Chromatographic separation was achieved with Agilent Poroshel 120 C18 column (2.1 mm × 75 mm × 2.7 µm), which was maintained at 30°C temperature. The HPLC mobile phases were 0.1 % formic acid in water (A) and 0.1 % formic acid in acetonitrile (B). An isocratic flow was maintained with 50 % mobile phase A and 50 % mobile phase B for 5 minutes. The total run time for analysis was 5 minutes. The mobile phase flow rate was maintained at 0.5 mL/min.

#### 2.5.2 Mass spectrometric (MS) conditions

Agilent 6460 Triple Quad QQQ with an electrospray ionization (ESI) source was applied for detection and quantification purposes. The multiple reaction monitoring (MRM) optimization option was then used to optimize MS parameters. The instrument control software Mass Hunter optimized the yield from collision energy. The mass spectral analysis selected a positive ionization (ESI) mode. MS source optimization parameters are summarized in Table 1.

**Table 1:** Mass spectrometry (MS) source parameters used for the analysis of glucocorticoid content in quackery formulations.

**Parameters**	**Optimization Values**
Scan Type	MRM
Polarity	Positive
Gas Temperature	350°C
Gas Flow	9 L/min
Sheath Gas Temp	350°C
Sheath Gas Flow rate	10 L/min
Nebulizer pressure	35 Psi
Capillary Voltage	4000 V

### 2.6 Method validation

The method was validated for analytical measurement range (AMR), carryover, selectivity, dilution effects, linearity (calibration curve) with recovery, a lower limit of quantification (LLOQ), accuracy, precision, sensitivity, stability, and dilution effects according to US Food and Drug Administration (FDA) guidelines (2018 guidance for industry) [26], [27]. The selectivity was investigated by analyzing blank and analyte standards. The precision and accuracy were assessed at four concentration levels (7.8, 31.25, 500, and 1000 ng/mL) according to FDA guidelines for method validation. All the samples were performed in triplicates.

### 2.7 Application for screening of glucocorticoids in quackery formulation powder

The validated method was applied to determine studied glucocorticoids in forty (40) quackery formulation powders that were randomly purchased from local markets in Pakistan.

## 3 Results and discussion

### 3.1 MS/MS condition optimization

Table 2 shows the MS acquisition parameters, including precursor ion, product ion, collision energy, dwell time and collision acceleration for each glucocorticoid. The analysis was performed using LC-MS/MS, with three independent replicates for each glucocorticoid to ensure reproducibility. Data were analyzed using Mass Hunter Software in MRM mode.

**Table 2:** Acquisition parameters for the quantification of Betamethasone, Dexamethasone, Hydrocortisone, and Prednisolone using LC-MS/MS.

**Compound**	**Precursor Ions (*m/z*)**	**Product/daughter Ions (*m/z*)**	**Dwell Time (m/sec)**	**Fragmentor(volt)**	**Collision Energy (volt)**	**Collision Acceleration(volt)**	**Polarity**	**Application**
Betamethasone	393.3	373.2	50	77	8	4	Positive	Quantitative
	393.3	337.2	50	77	16	4	Positive	Qualitative
Dexamethasone	393	355	50	77	8	4	Positive	Quantitative
	393	147	50	77	16	4	Positive	Qualitative
Hydrocortisone	363	121	50	77	16	4	Positive	Quantitative
Prednisolone	361	343	50	77	8	4	Positive	Quantitative
	361	307	50	77	16	4	Positive	Qualitative

**Fig. 3:**
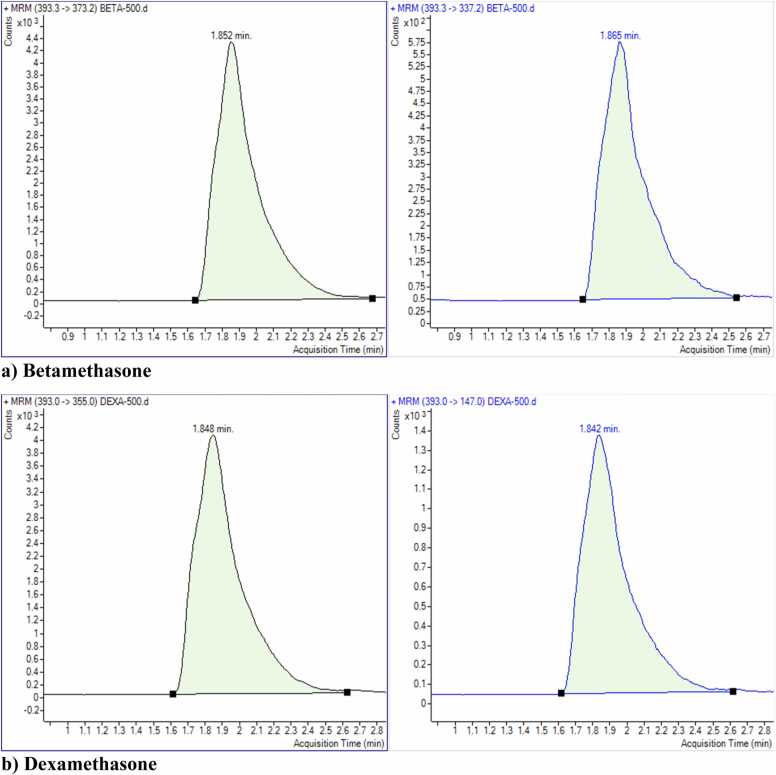

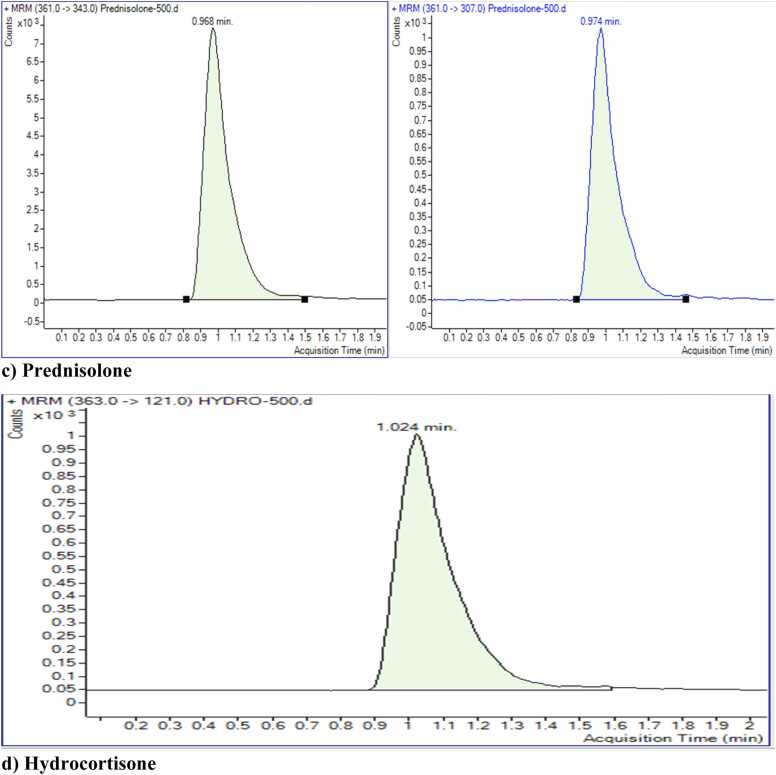
Representative chromatograms of four glucocorticoids, betamethasone, dexamethasone, hydrocortisone, and prednisolone, at a concentration of 500 ng/mL in methanol, analyzed using LC-MS/MS with an electrospray ionization (ESI) source.

Chromatograms of four glucocorticoids are shown in Fig. 3. Each chromatogram shows a clear separation of peaks under the optimized conditions. Betamethasone, dexamethasone, and prednisolone have been optimized with one precursor and two product ions. In contrast, hydrocortisone has been optimized with one precursor ion and one product ion. The molecular mass selects the precursor ion as [M+H]^+^. One transition was for quantitation, and the other was for confirmation.

Three solvents, i.e., acetonitrile, methanol, and water, were chosen for sample solution preparation of the formulating powders. Using the earlier sample preparation procedure, one gram of the sample was weighed and dissolved in 4 mL methanol. The same procedure was repeated for acetonitrile and water. The prepared sample solutions in different solvents were injected into LC-MS/ MS. Percentage recovery of the analyte in each solvent was calculated. For this, a known amount of analyte standard was added to each solvent and analyzed before and after spiking. The % recovery was highest for methanol (97.8 %), followed by acetonitrile (96.8 %), and then for water (95.6 %). Therefore, methanol was selected to extract four glucocorticoids in quackery formulation powder. The extraction efficiency of methanol has already been investigated in the literature for glucocorticoids [1], [4].

### 3.2 Method validation

**Fig. 4:**
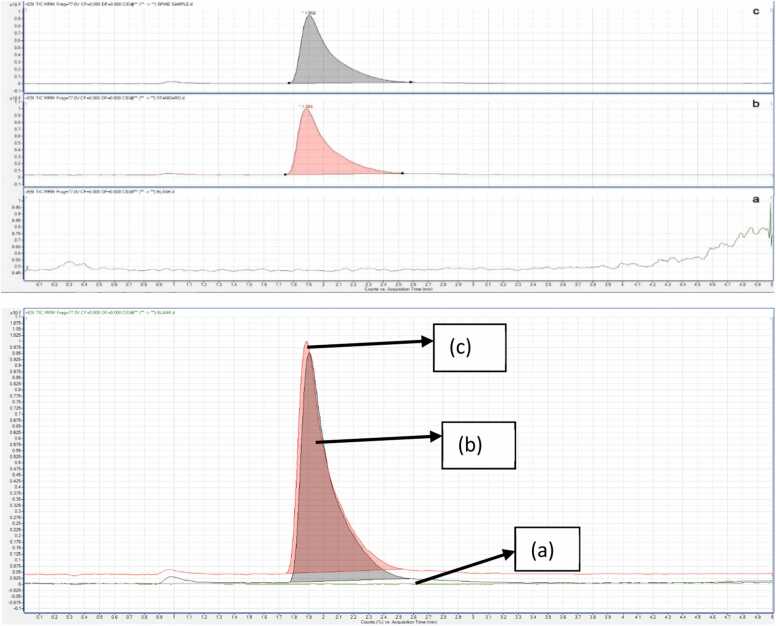
Representative chromatograms showing (a) the blank sample matrix without dexamethasone, (b) dexamethasone standard at a concentration of 500 ng/mL, and (c) a sample spiked with dexamethasone at 500 ng/mL.

The method selectivity was investigated by the chromatograms illustrated in Fig. 4 obtained from the blank, analyte standard, and spiked sample.

The blank chromatogram does not show any peak or baseline distortion near the retention time of the analyte. Similarly, the selectivity of betamethasone, hydrocortisone, and prednisolone was also investigated.

**Fig. 5:**
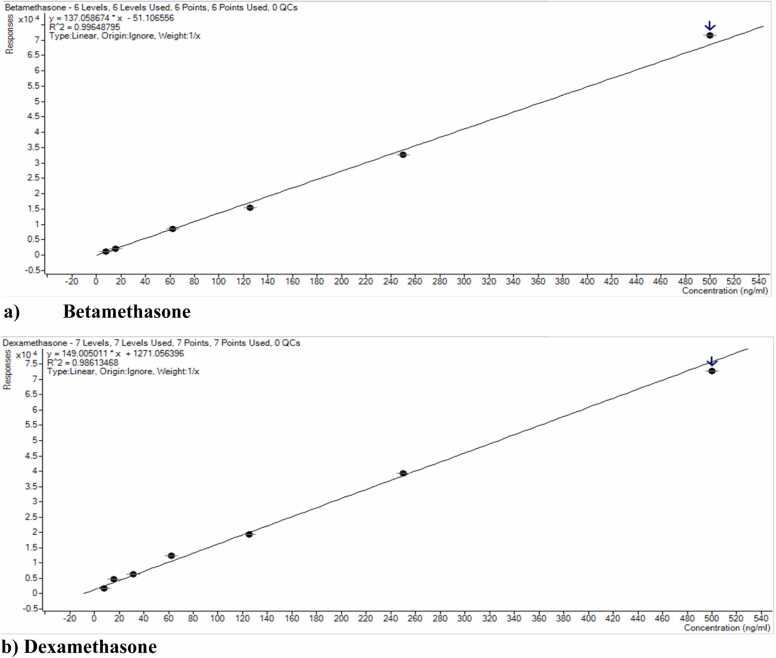

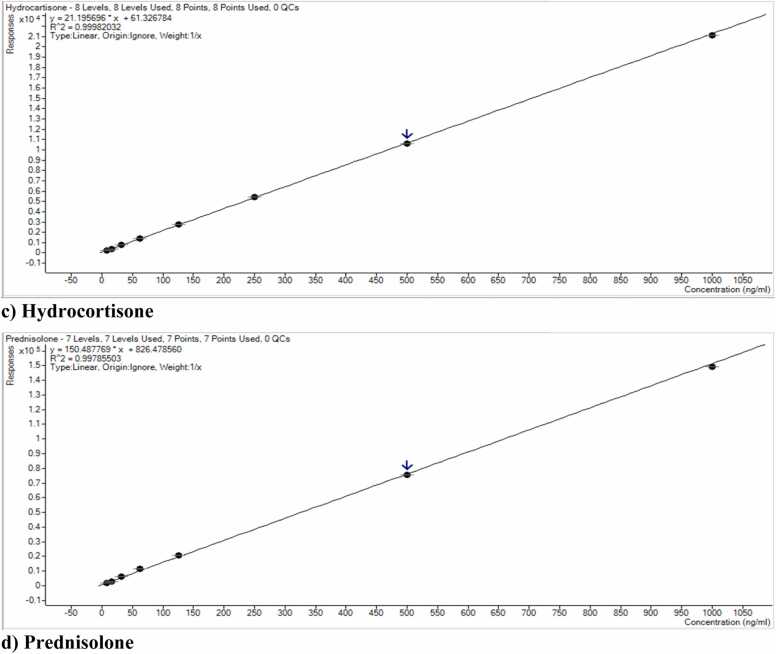
Calibration curves of (a) Betamethasone, (b) Dexamethasone, (c) Hydrocortisone, and (d) Prednisolone within the concentration range of 7.8 – 1000 ng/mL.

In Fig. 5, linear regression investigated linearity per FDA guidelines using six to eight calibration points throughout the operating range with a weight factor of 1/x. The equation for the straight line was used to calculate the slope and intercept, while the correlation coefficient (r^2^) was calculated from regression analysis. A linear relationship was found between analyte concentration and peak areas (concentration detected) throughout the AMR of 7.8 – 500 ng/mL for betamethasone and dexamethasone and 7.8 –1000 ng/mL hydrocortisone and prednisolone with coefficients of determination (r2) higher than 0.990 for betamethasone, hydrocortisone and prednisolone while for dexamethasone 0.986. The parameters of the calibration curves for betamethasone, dexamethasone, hydrocortisone, and prednisolone and the corresponding regression coefficients are summarized in Table 3. Fig. 5 shows the calibration curves of (a) Betamethasone, (b) Dexamethasone, (c) Hydrocortisone, and (d) Prednisolone in methanol, using 6–8 calibrators over a concentration range of 7.8 – 1000 ng/mL. Each calibration curve was generated from three independent replicates, and the error bars represent the standard deviation of these replicates.

**Table 3:** Linear regression data (using a 1/x weighting factor) for four glucocorticoids detected in quackery formulations.

**Sr no.**	**Compound**	**Linearity Range (ng/mL)**	**LLOQ Precision (%CV)**	**LLOQ Accuracy (%Bias)**	**Calibration Parameters**	
					**Regression equation**	**r**^**2**^
**1**	Betamethasone	7.8–500	0.9	−6.6	y=137.05*x−51.10	0.9964
**2**	Dexamethasone	7.8–500	8.2	10.6	y=149.00*x+1271.05	0.9861
**3**	Hydrocortisone	7.8– 000	7.05	6.8	y=21.19*x+61.32	0.9998
**4**	Prednisolone	7.8–1000	8	14.87	y=150.48*x+826.47	0.9978

The lower limit of quantitation (LLOQ) for selected glucocorticoids was determined as 7.8 ng/mL. The sensitivity was low enough to determine glucocorticoids in quackery formulation powder, where the typical concentration exceeds 7.8 ng/mL. The concentration of glucocorticoids in quackery formulation powders was mainly in the range of 10–1000 ng/mL.

Accuracy and precision of each selected glucocorticoid were studied by using four concentration levels; lower limit of quantitation (LLOQ), Low (L) (3 times LLOQ), Medium (M), and High (H). For each level, five replicates of the run were performed. For all glucocorticoids, the LLOQ was 7.8 with a bias of 6–15 % (<20 %) and precision of <15 %, as mentioned in Table 4.

**Table 4:** Accuracy and precision of four glucocorticoids (betamethasone, dexamethasone, hydrocortisone, and prednisolone) assessed through multiple replicate analyses using LC-MS/MS.

**Sample**	**Nominal value (ng/mL)**	**Accuracy**		**Intra-day and inter-days precision**			
		**Average (ng/mL)**	**Bias (%)**	**Mean (ng/ mL) Intra-day**	**Intra-days CV (%) n=10**	**Mean (ng/mL) Inter-days**	**Inter-days CV (%) n= 15**
**Betamethasone**							
LLOQ	7.8	8.37	−6.6	8.37	0.9	8.98	4.2
Low	31.25	32.12	−2.78	32.12	5.2	33.2	4.8
Mid	250	244	2.4	243.5	0.66	240.6	0.56
High	500	522.7	−4.54	522.7	1.5	517.2	1.3
**Dexamethasone**							
LLOQ	7.8	6.97	10.6	6.97	8.2	6.99	6.9
Low	31.25	34.4	−10.08	34.4	8.7	33.7	7
Mid	250	244	2.4	244	0.36	241	0.37
High	500	483	3.4	483	1.5	481.6	1.2
**Hydrocortisone**							
LLOQ	7.8	7.25	7.05	7.25	6.8	7.0	7.5
Low	31.25	32.82	−5.02	32.82	8	33.42	9
Mid	500	498.2	0.36	498.2	1	497.5	2.9
High	1000	994.1	0.59	994.1	1.8	995.3	1.5
**Prednisolone**							
LLOQ	7.8	6.64	14.87	6.64	8	6.55	5.7
Low	31.25	35.3	12.96	35.3	8.35	34.6	8.1
Mid	500	495.4	0.88	495.4	0.47	498	0.42
High	1000	986.9	1.31	986.9	0.78	987	0.77

All the glucocorticoids matched the acceptance criteria for accuracy as assessed by percent recovery at all four concentration levels (±15 %), as shown in Table 4.

In all concentration levels of four glucocorticoids, the precision was ˂15 % intra and inter-day. There were no distortions in either peak or baseline in the blank chromatogram. There was no interference among the four glucocorticoids, and their chromatograms were excellent, even at LLOQ. Moreover, there was no carryover since the response in blank was less than ±20 % of LLOQ. Hence, the method was suitable for quantifying glucocorticoids in quackery formulation powder.

### 3.3 Analysis of quackery formulation powders

Forty quackery formulation powders that were randomly purchased from local markets in Pakistan were used to evaluate the presence of glucocorticoids. Out of 40 quackery formulation powders, 23 (57.5 %) samples were contaminated with glucocorticoids, as mentioned in Table 5. The results of 23 quackery formulation powders contaminated with glucocorticoids indicated that 9 (39.1 % of 23 samples) contained dexamethasone, out of which 3 (3 out of 9; 33.3 %) also had prednisolone in addition to dexamethasone, 7 (30.4 %) betamethasone, 5 (21.7 %) prednisolone out of which 3 (3 out of 5; 60.0 %) were also contained dexamethasone in addition to prednisolone, and 2 (8.7 %) hydrocortisone. The concentration of glucocorticoids in all samples ranges from 10 to 1000 ng/mL. The distribution of glucocorticoids in QF powders is shown in Table 5.

**Table 5:** Distribution of Glucocorticoids in Quackery Formulating Powders, based on the analysis of three replicates using LC-MS/MS method.

**Samples taken**	**No. of uncontaminated sample**	**No. of samples contaminated with glucocorticoids**				**Total n (%)**
**Source of QF powder**		**Prednisolone**	**Dexamethasone**	**Hydrocortisone**	**Betamethasone**	
Unregistered Hakeems	17 (42.5 %)	5 (21.7 %)	9 (39.2 %)	2 (8.7 %)	7 (30.4 %)	23 (100 %)
**Total n (%)**	17 (42.5 %)	23 (57.5 %)				40 (100 %)

Our developed LC-MS/MS method is sensitive and has acceptable accuracy and precision. We recommend the use of this method for detecting, quantifying, and confirming the concentration of glucocorticoids in quackery preparations. We understand that better and more sensitive methods might be developed that serve the same purpose, which we are aiming for with better results. However, keeping our country's socioeconomic and laboratory facilities in perspective, this method would be the most readily implementable one, which was also one of the reasons for us to develop a method on LC-MS/MS. Among 40 studied quackery formulations, 23 (57.5 %) tested positive for glucocorticoids. This is a higher percentage than a previous study conducted in Pakistan by Syed Tanveer *et al.,* [29] using the HPLC technique, which stated that 21 % of quacks formulations collected from patients showed glucocorticoid contamination. According to WHO, 10–30 % of pharmaceutical preparations are counterfeit based on their contents. In China, about 14.1 % of tested samples were substandard [25], [30]. A much higher percentage (57.5 %) has been noted in our study.

Although LC-MS/MS has been reported for the detection of glucocorticoids in biosamples such as eggs and milk [31], bovine tissues [32] and cosmetics [1]; however, for powdery formulations, no literature is available using LC-MS/MS for the detection of glucocorticoids. Recently, Anwar *et al.,* [33] used the HPLC-UV method to evaluate steroidal contaminants in herbal medicines in Pakistan. Their method showed the linear assay of 0.6–1.4 μg/mL for dexamethasone, prednisolone, and betamethasone, which is much higher than the AMR of this developed method. At the same time, their reported LLOQ were 0.06, 0.192, and 0.438 μg/ mL for prednisolone, dexamethasone, and betamethasone, respectively, are significantly higher than those achieved by this developed method.

Similarly, Bu XM et al., [34] developed the MALDI-HRMS method for detecting androgenic steroids, which relies on d0/d5-Girard's reagent P labeling to enhance detection. Our developed method avoids this additional chemical labeling step and reduces preparation time, which contributes to practicality in routine analysis. Additionally, the LC-MS/MS method requires fewer specialized consumables compared to the MALDI-HRMS method, making it a more cost-effective and efficient solution for analyzing glucocorticoid content in powder formulations. Furthermore, the reported LLOQ (180–480 ng/mL) of the MALDI-HRMS method for androgenic steroids is significantly higher than our method, underline the enhanced sensitivity of our approach.

Comparison of the LC-MS/MS methods used for the detection of glucocorticoids (Table 6) shows that our developed method for detecting glucocorticoids in powder formulations demonstrates a significantly lower LLOQ (7.8 ng/mL) and shorter run time (within 2 minutes) compared to previous methods reported in chicken tissue [35], environmental water [36], and traditional Chinese medicines [34]. The detection sensitivity and speed of our method make it advantageous for routine analysis. Additionally, while the human plasma [37] method achieves a lower LLOQ (2 ng/mL) for betamethasone, its focus is limited to a single compound, whereas our method covers multiple glucocorticoids.

**Table 6:** Overview of various reported LC-MS/MS methods for the quantification and identification of steroids and glucocorticoids, including sample types, experimental conditions, linear assay, LLOQ, run time and analytical techniques used.

**Year**	**Sample type**	**Technique used**	**Number / Name of analytes**	**Extraction technique**	**Linear Assay**	**LLOQ**	**Run Time**	**Reference**
2019	Chicken Tissue	HPLC-MS/MS	40 glucocorticoids and 9 non-steroidal anti-inflammatory drugs (NSAIDs)	Solid Phase Extraction	2–100 μg/L	0.3–1.5 μg/kg	16 min	35
2020	Environmental water	HPLC-MS/MS	7 sex hormones	Solid Phase Extraction	10–150 μg/L	0.5–3.0 ng/L	18 min	36
2023	Human plasma	HPLC-MS/MS	Betamethasone	Liquid-Liquid Extraction	2–250 ng/mL	2 ng/mL	6 min	37
2023	Traditional Chinese medicines	HPLC-MS/MS	Androgenic steroid	d_0_/d_5_-Girard's reagent P labelling	0.20–200.00 μg/g	180–480 ng/mL	Not mentioned	34
2024	Quackery formulating powders	HPLC-MS/MS	dexamethasone, hydrocortisone, prednisolone, and betamethasone	Liquid-Liquid Extraction	7.8–1000 ng/mL	7.8 ng/mL	Within 2 min	Present work

This study intends to highlight the dangers associated with the intake of these quackery formulations and to bring up these observations in front of relevant authorities in order to help curtail this fraudulent practice in our country. We aim to disseminate this method to multiple testing facilities where it could be used on samples from different prescribers and distributors so that detailed observations can be made and appropriate actions are taken accordingly. Limitations of the study are that we have just validated the method for determining different glucocorticoids in quackery formulation. Although the comparison of methods has not been performed due to the lack of local availability of comparable approaches, this LC-MS/MS technique provides a robust and accessible alternative for detecting glucocorticoids in powder formulations, particularly in unregulated quackery formulations that pose significant health risks. Its sensitivity, specificity, and reproducibility offer reliable detection in a context where traditional methods may fall short, addressing critical gaps in regional analytical capabilities and underscoring the need for this approach.

Furthermore, we have only developed a method on LCMS-MS for different glucocorticoids in quackery formulation. We didn't use this method on human specimens like serum and urine. We recommend conducting further studies to validate the use of this method for determining glucocorticoid levels in the serum or urine of patients using these powders for various treatments, by which further detrimental effects of using them can be highlighted.

## 4 Conclusion

An LC-MS/MS method has been developed and validated for simultaneously determining levels of four glucocorticoids in quackery formulation powders. All the requirements of FDA guidelines for method validation have been successfully met. The method consists of sample extraction with methanol, followed by centrifugation and filtration. LLOQ of this method was enough to determine glucocorticoids in these powders. Consequently, the LC-MS/MS method successfully screened, confirmed, and quantitated glucocorticoids in these powders purchased from local vendors or quacks in Pakistan. The results indicated that 57.5 % of the tested samples detected glucocorticoids.

## CRediT authorship contribution statement

**Muhammad Irfan Jalees:** Writing – review & editing, Formal analysis, Data curation. **Hafiza Monaza Batool:** Writing – review & editing, Writing – original draft, Visualization, Validation, Software, Methodology, Investigation, Formal analysis, Data curation, Conceptualization. **Madeeha Batool:** Writing – review & editing, Visualization, Supervision, Resources, Project administration, Methodology, Investigation, Conceptualization.

## Declaration of Competing Interest

The authors declare that they have no known competing financial interests or personal relationships that could have appeared to influence the work reported in this paper.

## Data Availability

No data was used for the research described in the article.
